# Treatment outcome reporting in anorexia nervosa: time for a paradigm shift?

**DOI:** 10.1186/s40337-018-0195-1

**Published:** 2018-05-07

**Authors:** Stuart B. Murray, Katharine L. Loeb, Daniel Le Grange

**Affiliations:** 1 Department of Psychiatry, University of California, San Francisco, 401 Parnassus Avenue, San Francisco, CA 94118 USA; 2 School of Psychology, Fairleigh Dickinson University, Teaneck, NJ USA; 3 Department of Psychiatry and Behavioral Neuroscience, The University of Chicago, Chicago, IL USA

**Keywords:** Anorexia nervosa, Treatment outcomes, Eating disorders, Precision treatment

Anorexia nervosa (AN) is among the most pernicious of psychiatric disorders, demonstrating a mortality rate six times greater than the general population, and a crude mortality rate of 5–6% [1]. Even in non-lethal presentations, AN frequently runs a chronic and relapsing illness course, which imparts multi-systemic organ damage, including cardiac abnormalities, structural and functional brain impairment, and bone disease [2]. Alongside these grave medical sequelae, treatment outcomes in AN are universally modest. End-of-treatment remission rates in adolescent AN, the most common period of illness onset, are reported to range from 23 to 33% [3, 4], of which approximately one third remain in remission at four-year follow-up [5], whereas adult presentations are characterized by end-of-treatment remission rates ranging from 0 to 25% [6]. The urgent need for novel interventions for AN, as well as augmentations to the potency of existing treatment models, cannot be disputed.

As treatment development studies aim to illustrate and engage specific mechanisms of AN psychopathology, a crucial endeavor lies in precisely indexing the mechanisms of existing treatments, as they relate to the array of symptoms encompassed by AN, including physiological, cognitive and behavioral symptoms. However, existing methods for reporting treatment and course-of-illness outcomes in AN research may have precluded a thorough investigation of both treatment efficacy and mechanisms. Here we outline several important challenges relating to conceptualizations of outcome in AN, which must be considered as we advance towards the development of novel interventions with precise, targeted mechanisms.

## The weight- versus cognitive-symptom outcome conundrum

The symptom profile in AN comprises both physiological and cognitive features, although the central distinction between weight-based versus cognitive symptomatology has historically been underreported in AN treatment trials and long-term course-of-illness studies, with weight status alone being the most widely favoured index of recovery. In studies adopting weight as an exclusive or primary metric of outcome, potentially important differences may go undetected, as may their implications for our understanding of both disorder- and treatment-specific mechanisms. Without question, an essential first step in the treatment of AN is the reversal of the acute effects of starvation, which may be most readily indexed by weight status. However, inferring from positive increments in our patients’ weight that we have effectively engaged the target mechanism of treatment and achieved change in corresponding broader symptom domains, belies the complex and interwoven network of maintaining factors that underpin AN. Moreover, the implicit assumption that weight-based recovery is a proxy for broader cognitive recovery is not supported by evidence, as the constellation of cognitive and affective challenges facing those with AN, including the fear of weight gain, body dissatisfaction, emotional dysregulation, and an ongoing fear of calorie-dense foods, frequently persist after significant weight gain has been achieved [7]. Thus, relying on weight outcomes alone in drawing conclusions from randomized controlled trials (RCT) or course-of-illness studies could inadvertently inflate the interpretation of positive results. For instance, long-term naturalistic follow-up data over approximately 20 years illustrate remission rates ranging from 62% when considering weight status as the sole criterion for a ‘good outcome [8], to just 40% when including both weight and cognitive symptoms [9].

More recently, an increasing number of clinical trials have begun to report treatment outcomes as an aggregated function of both weight status *and* cognitive AN psychopathology, yielding categorical outcome groupings. For instance, “full” remission is typically achieved by attaining both (i) 95% of expected body weight plus (ii) a score within 1 standard deviation of community norms on gold standard measures of cognitive and behavioural ED psychopathology; “partial” remission can be defined by meeting either of those criteria, but not both; and no remission would reflect an absence of the two criteria. While this approach represents an improvement over exclusively weight-based outcomes, there are potential discrepancies between these component dimensions. Conflating weight-based and cognitive symptom status into unitary outcome measures represents a missed opportunity to elucidate their distinct pathways, which in turn can stymie ongoing attempts to locate precise mechanisms of treatment. Moreover, varying definitions of what constitutes a ‘good outcome’, even when applied to a single clinical trial, yields remission rates ranging from 2% - 96% [10], which has precluded meaningful between-trial comparisons.

## Moving forward: Key recommendations

Accurate indices of potentially differential treatment dimensions, rather than conflated unitary outcomes, are necessary to more closely understand treatment mechanisms. We contest that such delineated outcomes ought to be deemed equally primary to the results and discussion of controlled treatment trials as conflated categorical outcomes, which offer a broad snapshot of outcome. For instance, an RCT may find a significant superiority of one treatment over another in terms of categorical outcomes, yet fail to demonstrate any significant differences across the same treatments in delineated indices of weight or cognitive symptomatology [4]. Interpreting such patterns of results is an important initial step in the development of more potent precision interventions, and is predicated on RCTs systematically reporting results with multiple, and consistent, definitions of outcome. To date in the literature, only a subset of AN studies have done so.

Fully delineating indices of weight- versus cognitive AN symptomatology may not only foster a more nuanced understanding of their respective and combined patterns of change, but also the temporal relationship between them. For instance, examining the latency between nutritional rehabilitation and cognitive relief as a function of moderators, such as duration of illness, will direct our understanding of the intersection between chronicity, the biological effects of starvation, and the mechanistic underpinnings of cognitive AN symptomatology. Alongside these delineated outcomes, incorporating behavioural indicators and parent-report observations of cognitive symptoms will be critical to avoid false negatives in this type of research involving child and adolescent AN populations with ego syntonic presentations [11].

Finally, rigorous, detailed reporting of longer-term outcomes, as well as treatment engagement during follow up, will allow for more controlled tracking of symptom trajectories and pathways over time. Approximately half of RCTs for AN have reported long-term follow-up data, and of those that do, few have reported whether participants were engaged in ongoing treatment further to completion of the clinical trial, and whether ongoing treatment was concordant or discrepant from the intervention received during the RCT. Ongoing treatment-seeking will by definition be correlated with end-of-treatment symptom status; this means it does not occur at random, and thus represents a confound that compromises the validity of the field’s sparse longer-term data on AN.

Clearly, the development of novel treatments for AN is indicated, although a shift in our conceptualization of treatment outcomes may help identify precise targets for novel interventions. Notably, this may extend beyond weight status and diagnostic cognitive symptoms, i.e., shape and weight disturbance, to include other more transdiagnostic mechanisms such as intolerance of uncertainty and even purported endophenotypic features such as cognitive rigidity [12, 13]. To this end, establishing a benchmark that all treatment studies report independent effect sizes for both weight- and cognitive-based AN symptomatology, alongside indices of the proposed target mechanism of treatment, and details of ongoing treatment engagement among those included in follow-up data, will be a crucial step as the field transitions toward the development of precision treatments. In addition, the field’s adherence to an agreed-upon outcome reporting framework that incorporates these recommendations will also facilitate between-trial comparisons, and in turn, more informed practice guidelines for AN.
